# Outcomes of furcal perforation management using Mineral Trioxide Aggregate and Biodentine: a systematic review

**DOI:** 10.1590/1678-7757-2022-0330

**Published:** 2022-12-02

**Authors:** Saad Al-Nazhan, Iman El Mansy, Nada Al-Nazhan, Nbras Al-Rowais, Ghalia Al-Awad

**Affiliations:** 1 Riyadh Elm University College of Dentistry Restorative Dentistry Department Riyadh Saudi Arabia Riyadh Elm University, College of Dentistry, Restorative Dentistry Department, Riyadh, Saudi Arabia.

**Keywords:** Biodentine, Furcal perforation management, MTA, Outcome

## Abstract

Furcal perforation is an iatrogenic or pathologic communication between the pulp chamber floor and the alveolar bone. The outcome of perforation sealing depends greatly on the tissue compatibility and bioactivity and sealing properties of the repair materials. Mineral trioxide aggregate (MTA) and Biodentine are currently the most used materials to treat this condition. The present systematic review aimed to report the treatment outcome of repaired furcal perforation using MTA and Biodentine and identify which material would yield a better outcome. Methodology: A comprehensive search was conducted using the PubMed database to identify experimental studies and case reports that describe treatment of furcal perforation. Studies and case reports that evaluated the outcome of repaired furcal perforations using MTA and Biodentine, published in English from 2018 to April 2022, were identified. Unavailable full texts were excluded. Results: Initial screening of 724 articles (670 studies and 54 case reports). After discarding the duplicated studies, we reviewed 50 studies, selecting 13 for abstract analysis. We retrieved and evaluated full texts of eight studies and five case reports. Both materials had an equivalent success rate in the first three months but by 12 months Biodentine performed better than MTA clinically and radiographically. Conclusions: Repair of furcal perforation with Biodentine yields a better outcome compared to MTA.

## Introduction

Endodontic perforation is one of the most common causes of endodontic failure (nearly 10%) of all failed cases.^1^ Communication between the root canal system and the periradicular tissues leading to inflammation, bacterial infection, bone resorption, and proliferation of epithelial tissue characterizes endodontic perforation.^2^ The condition can occur due to deep carious lesions or root resorption, and can arise during post space preparation or can be iatrogenic during endodontic treatment. Up to 29% of all endodontic mishaps were reported as an accidental perforation and 87% were in the pulp chamber of molars.^3^

Furcal perforations have worse prognoses than other locations,^4^ and lead to furcal bone loss and treatment difficulties due to the nature of the anatomic and topographic complexity of the area.^5^ Treatment delay can cause more complications leading to tooth loss.^6^ The size and location of the perforation is important in predicting the treatment outcome, and the most favorable prognoses are usually associated with a small perforation located above the coronal or apical level of the crestal bone.^2^

Immediate sealing of the perforation defect with a biocompatible material favors a positive outcome.^7^ The perforation defect can be repaired surgically or non-surgically.^8^ An ideal material for treating perforations should be biocompatible with the surrounding tissue, non-absorbable with excellent antimicrobial and sealing properties, and of adequate radiopacity.^9^

Biodentine and mineral trioxide aggregate (MTA) materials induce tissue repair. The adequate radiopacity of MTA and its high pH properties, demonstrated successful sealing ability, low solubility, tissue compatibility, ability to set in the presence of blood, and the ability to induce odontoblastic differentiation.^4,10,11^ Similarly, Biodentine has a short setting time, a high compressive strength, good tissue compatibility, and induces cell proliferation and biomineralization.^12,13^

Biodentine and MTA, which material would yield a better outcome? We conducted this systematic review to compare the tissue response following treatment of furcal perforations with MTA and Biodentine reparative materials.

## Methodology

This study was approved by the Ethics Committee of Riyadh Elm University, registration number FUGRP/2021/265/672 and IRB approval number FUGRP/2021/265/672/641.

An electronic search of the last 5 years in PubMed (MEDLINE) up to April 2022 was conducted to focus on recently published studies. The search terms used were combinations of the following: *Perforations and endodontics, furcation perforation, pulp floor, furcation perforation and repair, furcation perforation and mineral trioxide aggregate, furcation perforation and MTA*, and *furcation perforation and Biodentine*.

### Inclusion criteria

Human and animal studies *in vivo*

Case reports

Published between 2018 and 2022

Use of MTA and/or Biodentine material to repair the defect

Published in English

Exclusion criteria

Book, systematic review, *in vitro* (lab) studies and conference abstracts

Published in a language other than English

Published before 2018

Studies with incomplete text

The clinically formulated PICO question (Population, Intervention, Comparison, and Outcome) strategy was organized as follows: P = Endodontic treatment of patient or animal, I = Patient or animal with a perforation, C = Compare MTA and Biodentine, O = outcome of repaired furcation perforation.

Three independently trained final year dental students screened the full texts of the studies allocated to determine if they met the inclusion criteria. *In vivo* studies and case reports were included regarding the use of MTA or Biodentine materials to repair furcal perforations.

The identified studies were manually imported and screened for eligibility according to their title/abstract; duplicates were removed. Studies and case reports that met the inclusion criteria were included in the review. An expert endodontist supervised all steps of the search to ensure that the junior investigators reached a consensus.

The extracted data were entered into spreadsheet in Microsoft Office Word 2013 (Microsoft Corporation, USA). The research study data included authors, publication year, species, perforation size, sample size, type of material used, observation time, evaluation (diagnostic) methods, and outcome. The case report data included authors, publication year, patient age and sex, tooth number, cause of perforation, type of material used, observation time, evaluation (diagnostic) methods, and outcome. All results were presented as descriptive data only. SYRCLE’s risk of bias tool for animal studies as reported by Hooijmans, et al. ^14^ (2014) was followed.

## Results

**Figure 1:**
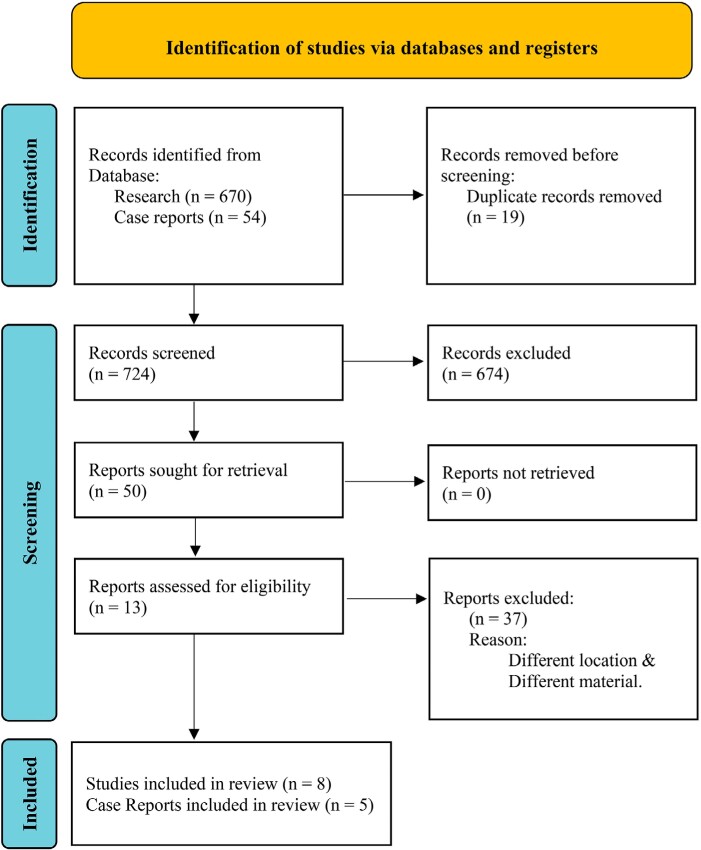
PRISMA flowchart of the screened and selected articles

Initial pre-screening of all databases yielded 724 articles (670 experimental studies and 54 case reports) in PubMed. We discarded the duplicates and we reviewed 50 articles, thus selecting 13 articles (eight studies and five case reports) for analysis of the abstract and retrieval of the full text (Figure 1).

**Figure 2:**
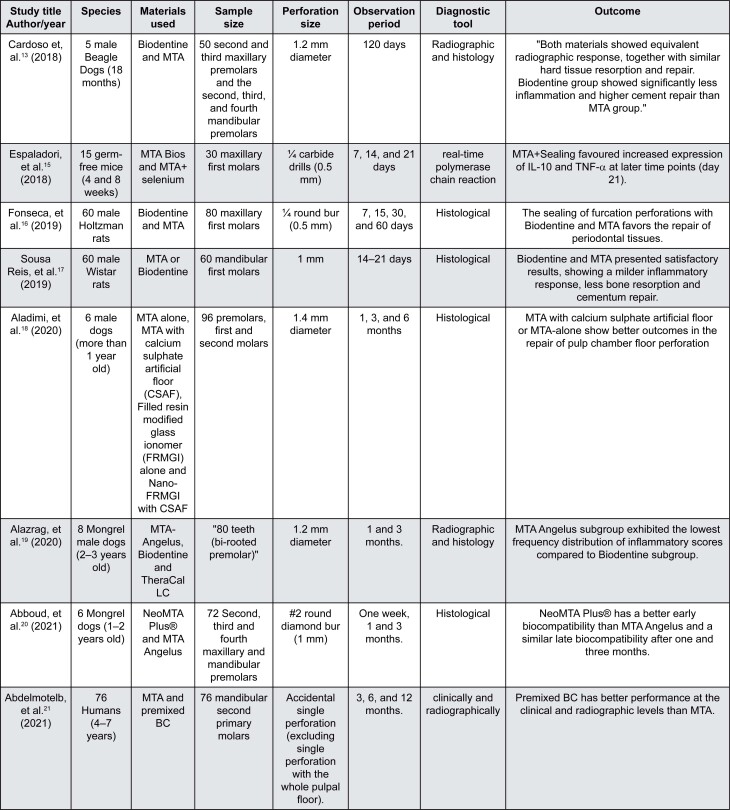
Summary of the characteristics and outcome of the included studies

**Figure 3:**
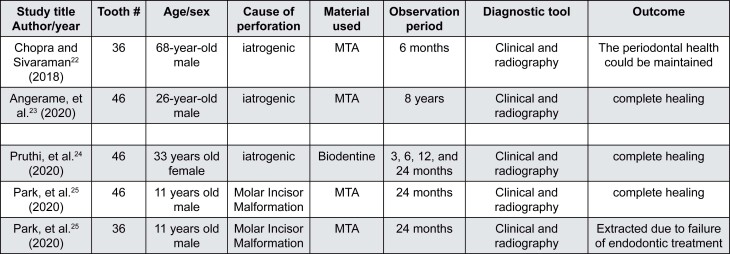
Summary of the characteristics and outcome of the included case reports

Figures 2 and 3 describes the main characteristics of the included studies and case reports. Biodentine and MTA were tested on animals and humans with equal sample sizes. The animal studies were evaluated histologically, whereas for humans clinical and radiographs were used. Both materials showed a satisfactory histological result. Cardoso, et al.^13^ (2018) reported no significant differences in hard tissue resorption between Biodentine and MTA. Moreover, Sousa Reis, et al.^17^ (2019) reported that both materials showed mild inflammatory response, less bone resorption and cementum repair. The clinical and radiographic evaluation showed better performance of Biodentine (Figure 2). Only one study that deals with humans did clinical evaluation by checking the sign and symptoms of the treated cases.^21^ The radiographic evaluation determined the presence, development or increase of radiolucency adjacent to the perforation site.^13,19,21^ The results of radiographic evaluation agree with those of the histological findings.^13,19^

We evaluated five case reports of furcal perforation treated with Biodentine and MTA clinically and radiographically (Figure 3). Complete healing occurred within 24 months. MTA was used on an 11-year-old male to treat right and left mandibular molars. Molar-incisor malformation affected both teeth. Complete healing occurred within 24 months on the right molar and the treatment failed on the left molar.

### Risk of bias in the studies included

**Figure 4:**
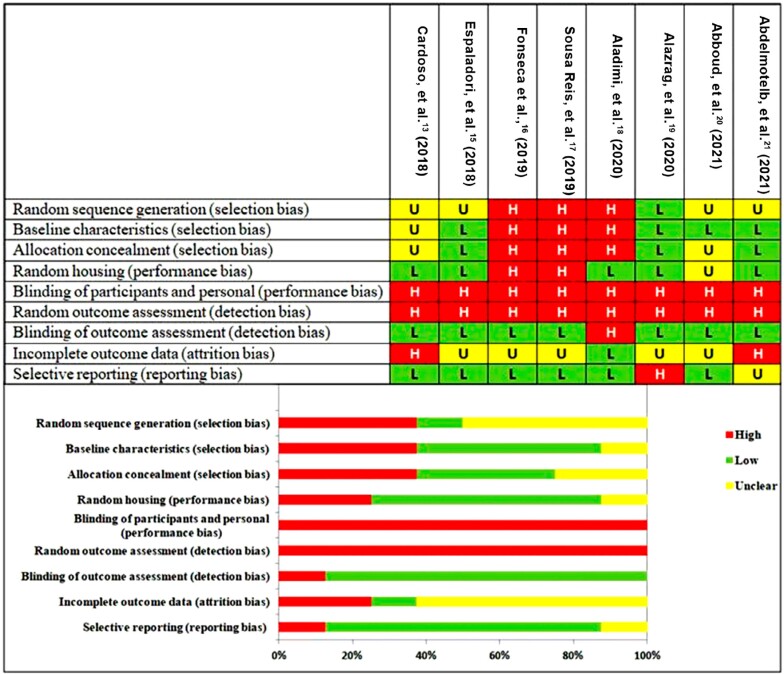
Risk of bias

All studies had a high risk of bias from the standpoints of blinding of participants and personnel and random outcome assessment. All eight studies were included. Three studies (40%) had a high risk of bias due to random sequence generation, baseline characteristics, and allocation concealment. Two studies had a low risk of bias due to blinding of outcome assessment and selective reporting. More than half of the studies had an unclear risk of bias due to incomplete outcome data; overall, the risk of bias in all the included studies was high (Figure 4).

## Discussion

By using the MEDLINE database, we performed the literature search for the current review. MEDLINE is the main subset of PubMed, in which researchers update online searches for research literature in the biomedical and life sciences, including health. The database is different from Scopus and the Web of Science, which embrace online journal articles. Moreover, searching is free of charge, does not require registration, and includes a link to the free full-text article. Google Scholar, in contrast, provides imprecise information for citations.^26^

We evaluated the success of Biodentine and MTA to repair furcation perforation in this review based on the search results from the past 5 years. Biodentine and MTA were tested on mice, rat, and dog models. Although few studies used rats, it is a suitable model histologically similar to dogs, the most used animal model for *in vivo* studies.^20,27,28^ The smaller mouth and size of the teeth of the rats complicates the clinical procedures compared to the use of dogs. However, the anatomy of the periodontium, histopathology of the periodontal lesions, and basic immunobiology of this model have been reported to be similar to those of humans,^27,29^ regardless of the non-comparable relationship between the bone margin and the furcation area in the dog model.^30^ Germ-free rats were used by Espaladori, et al.^15^ (2018) to avoid effects by indigenous bacterial microbiota.

MTA was the chosen material with an enhanced healing rate due to its good tissue compatibility and sealing ability.^10,31^ The results of the studies in the current review confirmed its role in healing the perforation defect (Figure 2). However, MTA’S poor handling and extended setting time have driven the use of other biomaterials such as Biodentine. Biodentine is biocompatible, similar to MTA, but does not contain bismuth oxide or calcium aluminate, which shortens the setting time compared to MTA.^32^

In the present review, analysis of the histopathological biopsies revealed that filling the perforation site with Biodentine or MTA promoted complete repair of the perforations due to a high frequency of mineralized tissue formation.^13,16,17^ Similar findings were reported by Al-Daafas and Al-Nazhan^33^ (2007) and Silva, et al.^34^ (2017).

Premixed Biodentine used to repair furcation perforation in human teeth performed better at the clinical and radiographic levels than MTA due to its ease of handling and sealing properties and its higher tissue compatibility, based on the results of the absolute risk difference of the clinical and radiographic success rates, calculated at a 95% confidence interval.^21^ The higher tissue compatibility of Biodentine over MTA was attributed to the absence of heavy metals found in MTA, which leach into bodily tissues and fluids.^35^ We believe that the small amount used to repair the perforation defect does not influence the healing rate.

*In vivo* animal studies are important prior to application in humans. Only one clinical trial used both materials to immediately manage furcal perforation.^21^ The clinical studies used radiological criteria to evaluate the affected area in addition to clinical observation.^36^ The reported human case in this review showed that Biodentine displayed better clinical and radiographic outcomes than MTA.^21^ This outcome was attributed to the superior sealing quality of Biodentine due to better handling and resistance to occlusal load, as well as the formation of dentinal tags observed by the scanning electron microscope, which provided strength for its dislocation.^37,38,39^ The formation of the dentinal tags was related to the calcium and silicon ion uptake into dentin.^40^ Moreover, Biodentine is more resistant to exposure to irrigating solutions.

Aside from the biological and physical characteristics of Biodentine and MTA, the perforation size of the furcal defect may influence the healing process. The size of perforations reviewed in animal studies ranged from 0.5 to 1 mm in rodents and 1 to 1.4 mm in dogs. However, a recent study concluded that perforation size has no influence on the treatment outcome,^41^ in contrast to the results of Askerbeyli Örs, et al.^42^ (2019). Conflicting results should be carefully considered due to variations in study designs, evaluation methodology, materials used, perforation size, tooth type, location, and follow-up period.

Meta-analysis could not be done due to variation in observation period evaluation and perforation size for each study investigated, as well as missing data related to the perforation size in the reported cases.

Extrusion of the repaired material into the surrounding tissues may compromise the outcome of furcal perforation repair. Artificial materials such as calcium sulfate have been used as a barrier to prevent material extrusion and epithelial migration into the defected perforation area;^33,43^ however, the use of barriers did not improve treatment outcomes.^33^

In the present review, we retrieved five clinical cases. We used Biodentine in only one case. Complete healing occurred in all cases except one. Pruthi, et al.^24^ (2020) successfully used platelet-rich fibrin as an external matrix in treatment of furcal perforation. It was placed in the perforated site then Biodentine was compacted over it. Complete healing of the defect was reported. They attributed this to the osteoconductive and osteoinductive properties of the resorbable platelet-rich fibrin tissue to enhance bone regeneration, resulting in accelerated wound healing.

The present review revealed that Biodentine and MTA performed similarly and yielded excellent treatment outcomes, regardless of differences in the experimental model. The reported clinical cases also noted this similarity.

This systematic review had some limitations. Only eight studies were retrieved due to the limited search methodology and search words used. Future work should expand the search using other search databases, such as Web of Science and Scopus.

## Conclusion

Despite the high risk of bias and the low number of the studies included, Biodentine yields a better outcome than MTA in the repair of furcal perforations.
